# Effects and Mechanisms of Kaempferol in the Management of Cancers through Modulation of Inflammation and Signal Transduction Pathways

**DOI:** 10.3390/ijms24108630

**Published:** 2023-05-11

**Authors:** Ahmad Almatroudi, Khaled S. Allemailem, Wanian M. Alwanian, Basmah F. Alharbi, Faris Alrumaihi, Amjad Ali Khan, Saleh A. Almatroodi, Arshad Husain Rahmani

**Affiliations:** 1 Department of Medical Laboratories, College of Applied Medical Sciences, Qassim University, Buraydah 51452, Saudi Arabia; aamtrody@qu.edu.sa (A.A.);; 2 Department of Basic Health Sciences, College of Applied Medical Sciences, Qassim University, Buraydah 51452, Saudi Arabia

**Keywords:** kaempferol, signal transduction, apoptosis, inflammation, cancer therapy

## Abstract

Cancer is the principal cause of death and its incidence is increasing continuously worldwide. Various treatment approaches are in practice to treat cancer, but these treatment strategies may be associated with severe side effects and also produce drug resistance. However, natural compounds have established their role in cancer management with minimal side effects. In this vista, kaempferol, a natural polyphenol, mainly found in vegetables and fruits, has been revealed to have many health-promoting effects. Besides its health-promoting potential, its anti-cancer potential has also been described in in vivo as well as in in vitro studies. The anti-cancer potential of kaempferol has been proven through modulation of cell signaling pathways in addition to the induction of apoptosis and cell cycle arrest in cancer cells. It leads to the activation of tumor suppressor genes, inhibition of angiogenesis, PI3K/AKT pathways, STAT3, transcription factor AP-1, Nrf2 and other cell signaling molecules. Poor bioavailability of this compound is one of the major limitations for its proper and effective disease management actions. Recently, some novel nanoparticle-based formulations have been used to overcome these limitations. The aim of this review is to provide a clear picture regarding the mechanism of action of kaempferol in different cancers through the modulation of cell signaling molecules. Besides this, strategies to improve the efficacy and synergistic effects of this compound have also been described. However, more studies are needed based on clinical trials to fully explore the therapeutic role of this compound, especially in cancer treatment.

## 1. Introduction

Worldwide, cancer is one of the major causes of death, with increasing incidence and mortality loads [1]. As per the 2018 GLOBOCAN database, approximately 18.1 million new cases of cancer and 9.6 million cancer deaths occur globally. The new cases of cancer will reach around 20 million by 2025 [2,3]. In the past few years, despite significant progress in cancer therapy, the specified incidence of cancer and mortality have not been reduced [4].

Many factors are involved in cancer development and progression including the alteration of cell signaling molecules. The mechanism of altered signal transduction pathways in this disease and the effects on apoptosis, tumorigenesis and metastasis have been extensively demonstrated owing to increasing research [5]. Various processes are involved in the progression of this disease and capacity of cancer cells to evade apoptosis [6], produce growth signals that are self-sufficient [7] and prompt angiogenesis [8]. 

The current modes of treatment approaches are effective but also cause toxicity and adverse effects to the normal cells. Henceforth, there is a need to devise alternative, safe and effective modes of treatment to tackle this disease without any adverse complications. Some plant extracts have shown minimal adverse effects as compared to chemotherapeutic drugs, and thus, currently, natural therapies are widely used as an alternative treatment [9]. In this regard, bioactive compounds from different natural products have shown a wide role in cancer management through modulation of various cell signaling pathways [10,11,12,13,14,15,16].

Flavonoids such as quercetin, apigenin, luteolin, vitexin and kaempferol are commonly distributed in foods and beverages of plant origin, including vegetables, fruits, tea, wine and cocoa. Its role in disease management including cancer has been described through the modulation of cell signaling pathways. In addition to influencing mammalian metabolism, flavonoids have been associated with a varied range of antiviral, anti-inflammatory, antiproliferative and anticarcinogenic properties [17]. Various clinical trial-based studies have reported flavonoids’ or polyphenol’s role in cancer treatment. One study conducted a placebo-controlled, randomized clinical trial of Polyphenon E (PolyE), a branded mixture of green tea catechins, comprising 400 mg (-)-epigallocatechin-3-gallate (EGCG) per day, in 97 subjects with atypical small acinar proliferation (ASAP and high-grade prostatic intraepithelial neoplasia (HGPIN)). Daily intake of a standardized, decaffeinated catechin mixture holding 400 mg EGCG per day for 1 year accumulated in plasma and was well-tolerated but did not reduce the likelihood of prostate cancer in men with baseline HGPIN or ASAP [18]. 

In a phase 2 trial for patients with chronic lymphocytic leukemia (CLL), it was found that once-daily oral administration of EGCG in the Polyphenon E formulation was safe and well-tolerated. Declines in ALC and/or lymphadenopathy were observed in the majority of patients [19]. A study aimed to evaluate the effectiveness of flavonoid fisetin supplementation on the inflammatory status and matrix metalloproteinase (MMP) levels in colorectal cancer patients. Conferring to the results, fisetin could improve the inflammatory status in colorectal cancer patients, suggesting it as a novel complementary antitumor agent for these patients [20]. Other results showed a substantial decrease in serum levels of prostate-specific antigen (PSA), vascular endothelial growth factor (VEGF) and hepatocyte growth factor (HGF) in men with prostate cancer after short-term treatment with EGCG (Polyphenon E), with no raise in liver enzymes. These findings back a potential role for Polyphenon E in the treatment or prevention of prostate cancer [21]. 

**Figure 1:**
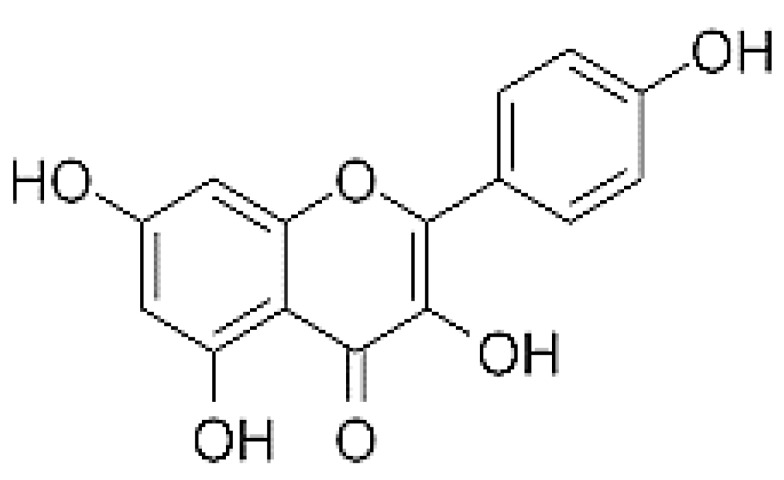
Chemical structure of kaempferol (3,4′,5,7-tetrahydroxyflavone), a flavonol found in different plants.

Kaempferol is a natural flavanol (Figure 1), mainly found in vegetables and fruits, and possesses a variety of therapeutic properties including antioxidant [22], anti-inflammatory [23] and anti-cancer effects [24]. Kaempferol and some of its glycosides can also meaningfully inhibit the production of nitric oxide (NO) as well as tumor necrosis factor alpha (TNF-α) in RAW 264.7 cells stimulated by lipopolysaccharides (LPS) [25]. An earlier study linking flavonoids with myocardial infarction and fatal coronary heart disease examined that kaempferol from broccoli and tea was inversely linked with coronary heart disease, whereas non-association was found with myocardial infarction [26]. The protective role of kaempferol against reactive oxygen species (ROS)-induced hemolysis and its antiproliferative activity on human cancer cells was examined. Kaempferol showed strong cellular antioxidant ability. Further, pretreatment with kaempferol meaningfully reduced the ROS-initiated hemolysis of human erythrocytes and decreased the accumulation of the toxic lipid peroxidation product malondialdehyde.

The anti-hemolytic potential of kaempferol mostly occurs via scavenging excessive ROS and preserving the intrinsic antioxidant enzyme activities at normal levels. Furthermore, kaempferol exhibited noteworthy antiproliferative activity on a panel of human cancer cell lines [27]. Furthermore, Kaempferol showed anti-cancer potential via numerous pathways including the inhibition of angiogenesis as well as the expression of VEGF, regulation of hypoxia-inducible factor 1-alpha (HIF-1α) apoptosis induction, induction of G2/M cell cycle arrest and caspase-3-dependent apoptosis [28,29]. Kaempferol could reverse the drug resistance of HCT8-R cells to 5-Fluorouracil (5-FU), demonstrating that kaempferol only or in combination with 5-Fluorouracil has the ability to treat colorectal cancer. Definitely, kaempferol treatment meaningfully decreased glucose uptake and lactic acid production in drug-resistant colorectal cancer cells. Finally, data propose that kaempferol may show a significant role in overcoming resistance to 5-FU therapy by controlling the miR-326-hnRNPA1/A2/PTBP1-PKM2 axis [30].

The bioavailability of kaempferol is reported to be very low. However, the nanoformulation-based studies have shown improvement in the efficacy of this compound. Moreover, kaempferol in combination with other anti-cancer drugs exhibits synergistic effects in the form of enhanced apoptosis and decreased cell viability in cancer cells. 

In addition to other findings, this review summarizes the anti-cancer potential and possible mechanisms of the action of kaempferol in numerous cancers via inhibition of angiogenesis [31], induction of apoptosis [32], activation of tumor suppression genes [33], STAT3 [34], AP-1 [35], P13K/Akt [36], cell cycle arrest [37] and other molecules [38]. Moreover, synergistic effects of kaempferol in combination with various anti-cancer drugs and strategies to improve the efficacy of this compound are described properly. The information compiled in this review will be helpful to the researchers to know the details about chemoprotective effects of this compound and its possible implication in clinical trials.

## 2. Mechanism of Action of Kaempferol in Cancer Prevention

**Figure 2:**
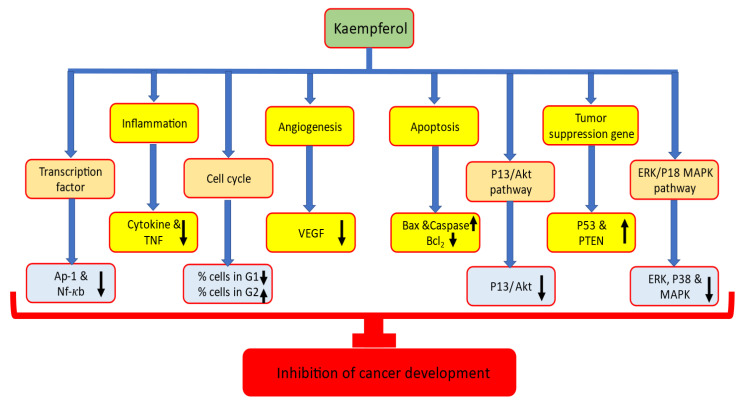
Mechanism of action of kaempferol in cancer prevention through inhibition of inflammation [39,40], angiogenesis [41,42], induction of apoptosis [43], activation of tumor suppressor genes [33], inhibition of transcription factors [34,44,45], inhibition of ERK/MAP pathway [46] and cell cycle arrest [29,47]. Up-arrow (↑) indicates up-regulation and down-arrow (↓) indicates down-regulation.

Kaempferol has proven to have substantial anti-cancer potential through modulation of cell signaling molecules [31,32,33,34,35]. The role of kaempferol in inhibiting various cancers along with its mechanism of action have been described below (Table 1 and Figure 2).

### 2.1. Inflammation

About 15–20% of all cancer cases are caused by chronic inflammation, infection or autoimmunity at the same tissue or at the site of disease [55,56]. In depth, chronic inflammation is involved in immuno-suppression, thus favoring a microenvironment for tumorigenesis, tumor development and metastasis [57]. In this view, natural compounds or bioactive ingredients play a vital role in disease management through their anti-inflammatory action. An important study based on cervical cancer reported that kaempferol restricts inflammation via suppressing the production of pro-inflammatory cytokines as well as chemokines. In contrast, kaempferol treatment at a 50 µM dose significantly upregulated anti-inflammatory cytokines such as IL-4, -10 and -13. 

Kaempferol restricts inflammation via suppressing the pro-inflammatory cytokines as well as chemokines production, such as M-CSF, IL-8, IL-7 and IL-16 MIG, that show borderline alteration, while TNF-β, s TNF RI, IL-1β, GM-CSF, MCP-1, MCP-2, TNF-α, s TNF RII, RANTES and EOTAXIN displayed substantial downregulation [58]. Single fluoxetine pre-treatment holds an important apoptotic effect via enhancing the activity of serum and colon tissue caspase 3. It also reduces the Dimethylhydrazine (DMH)-driven increase in colon tissue. It also attenuated the 1,2 Dimethylhydrazine (DMH) driven increase in Malondialdehyde (MDA, Nitric oxide, Proliferating cell nuclear antigen (PCNA) and Cyclooxygenase-2 (Cox-2) expression and serum as well as colon tissue β-catenin, with a decrease in the number of multiple plaque lesions (MPLs) and the multiplicity of aberrant crypt foci (ACF). The combination of fluoxetine with either kaempferol or epigallocatechin-gallate (EGCG), a chief ingredient of green tea, improved its anti-inflammatory, antioxidant and antiproliferating activities, with higher apoptotic activity observed in combination with kaempferol [39]. Kaempferol was capable of relieving the downregulatory potential of TNF-α on LXR-α in a dose-dependent fashion. The highest dose of kaempferol nearly stopped the action of tumor necrosis factor-α (TNF- α) by returning the levels of LXR-α to their basal levels in human hepatocarcinoma (HepG2) cells. Additionally, kaempferol (20 μM) alone did not show any substantial result on the expression of nuclear hormone receptor liver-X-receptor alpha (LXR-α) mRNA. Overall, the study established that kaempferol was able to ease TNF-α, which is an adipokine and a cytokine showing downregulatory effects on LXR-α mRNA in HepG2 cells [40]. The anti-inflammatory effect of kaempferol on lipopolysaccharide and ATP-induced cardiac fibroblasts was investigated. Findings revealed that kaempferol at concentrations of 12.5 to 25 μg/mL substantially suppressed the release of *TNF*-*α*, Interleukin-18 (IL-18), Interleukin-1β (IL-1β) and Interleuki-6 (IL-6) and inhibited activation of nuclear factor kappa B (NF-*κB*) and Akt [59].

### 2.2. Angiogenesis

Initiation of blood vessel formation is prompted when pro-angiogenic signaling is dominating, a process that in tumors has been coined as “angiogenic switch” [60]. Though, prevention/inhibition of the angiogenesis process is an imperative step in the direction of inhibition of cancer growth and advancement. Natural compounds have a proven anti-angiogenic role in tumor cells. Cell lines (OVCAR-3 and A2780/CP70) were treated with kaempferol and assayed to check VEGF mRNA and protein levels. Results revealed a downregulation of VEGF mRNA levels to 57% in OVCAR-3 cells and 72% in A2780/CP70 cells. Additionally, for 24 h kaempferol treatment (20 μM), VEGF mRNA expression was decreased to 73% and 81% in OVCAR-3 and A2780/CP70 cells, respectively. Both cell lines showed concentration-dependent inhibition on VEGF mRNA levels by kaempferol treatment. Moreover, VEGF (a potent angiogenic factor) and its protein levels in cell culture supernates were downregulated by kaempferol treatments [41].

Kaempferol meaningfully decreased VEGF-stimulated human umbilical vein endothelial cells’ (HUVECs) viability. Kaempferol treatment also inhibited tube formation in VEGF-stimulated HUVECs, cell migration and invasion. VEGF receptor-2 (VEGFR-2) and its downstream signaling cascades including MEK1/2-ERK1/2, AKT and mTOR were reduced. This finding showed that kaempferol might hold the inhibition of angiogenesis via control of VEGF/VEGFR-2 and its downstream signaling cascades (MEK, ERK and PI3K/AKT) in VEGF-stimulated endothelial cells [61]. It was recognized that kaempferol time-dependently inhibited VEGF secretion and suppressed in vitro angiogenesis. Kaempferol downregulated NF-κB and c-Myc expression and ERK phosphorylation, while it promoted p21 expression. Evaluation of the association between these genes proposed a novel ERK-NFκB-cMyc-p21-VEGF pathway, which is responsible for kaempferol angio-prevention activity in ovarian cancer cells [31]. An experiment was performed based on human ovarian cancer cells to evaluate the effects of different flavonoids and other substances on cell proliferation as well expression of VEGF. Results demonstrated that kaempferol, apigenin, quercetin, taxifolin, genistein and luteolin inhibited the ovarian cancer cell growth in a dose-dependent fashion [42].

### 2.3. Apoptosis

Failure to initiate the complete apoptosis in an unhealthy cell population is a reason that cells grow out of control, leading to cancer [62]. Recent studies confirmed that natural compounds show a vital role in cancer management through the inhibition of cancer cell proliferation, metastasis, induction of apoptosis and angiogenesis [63,64,65,66]. The kaempferol-induced apoptosis has been linked with the p53 upregulation. Additionally, the phosphorylation of p53 at the Ser-15 residue was noticed with kaempferol. In addition, kaempferol inhibits the proliferation of cells via disrupting the cell cycle, which is significantly related to the induction of arrest at the G2/M phase and might initiate apoptosis through p53 phosphorylation in breast cancer [32]. As kaempferol is cytotoxic to cancer cells, it was examined whether this was linked with the induction of apoptosis. Results demonstrated that kaempferol treatment prompted this apoptotic phenomenon in a time-dependent manner. Additionally, kaempferol treatment increases the population of annexin V-positive cancer cells. Moreover, the initiation of apoptosis was reduced by the treatment with z-VAD-fmk (a pan-caspase inhibitor). It was determined that the apoptotic response was facilitated by caspases and this finding revealed that kaempferol induced cell apoptosis [47]. 

A recent study reported that a significant increase in apoptosis in PANC-1 as well as Mia PaCa-2 cells occurs in response to 50 μM kaempferol treatment and the rate of apoptosis is positively increased with its concentration. Together, these results designate that Akt/mTOR signaling inactivation is involved in the kaempferol-encouraged apoptosis of pancreatic cells [67]. A leukemia-based study reported that kaempferol decreased the sub-G1 population and cell viability. This outcome was related to the reduced expression of Akt, Bcl2, ABCC1 and ABCB1 genes, whereas the expression of the BCL-2/ BAX ratio as well as CASP3 were suggestively superior than earlier at both gene and protein levels. Additionally, kaempferol prompted apoptosis and also minimized multi-drug resistance in a concentration-dependent fashion [43].

### 2.4. Tumor Suppressor Gene (PTEN)

Phosphatase and tensin homolog (*PTEN*) is one of the important tumor suppressor genes that shows a vital role in the inhibition of cell growth and increases the cellular sensitivity to apoptosis [68]. Altered *PTEN* function or loss of PTEN protein expression has been found in cancers [69]. Natural products play a significant role in cancer management through enhancement of PTEN gene/protein function. An important study based on bladder cancer was performed and levels of PTEN and Akt in cancer cells treated with kaempferol was evaluated. It was noticed that expression of PTEN increased in a time-dependent fashion in cancer cells treated with 40 μM of kaempferol, while Ser473-phosphorylated Akt expression was significantly reduced after treatment with same concentration of kaempferol. Together, these results showed that kaempferol inhibits pAkt Ser473 and upregulates PTEN expression in cancer cells [33].

### 2.5. Autophagy 

Autophagy is one of the evolutionary conserved cellular processes where proteins, lipids and organelles are broken down within lysosomes [70]. Additionally, autophagy is used for the elimination of other pathogens [71]. Autophagy depends on environmental conditions, such as tumor, stress type and stage, and it controls pro-survival or pro-death signaling pathways in various cancers [72]. In this regard, numerous natural compounds play a role in the modulation of autophagy. A study based on liver cancer reported that autophagic vacuoles were noticed when cancer cells were exposed with kaempferol. To evaluate whether exposure of kaempferol caused autophagy, cells were stained with Monodansylcadaverine (MDC) to detect acidic vesicular organelles’ (AVOs) formation. After kaempferol treatment, AVO-positive cells were greater than earlier in a concentration-dependent fashion as compared to the control. Based on these findings, it was determined that kaempferol encouraged autophagy [29]. Kaempferol showed a role in the promotion of autophagy and induces autophagic cell death in gastric cancer. Moreover, it was also noted that kaempferol caused the induction of autophagic cell death through initiation of the inositol-requiring-1 (IRE-1)-c-Jun N-terminal kinase (JNK)-C/EBP homologous protein (CHOP) signaling [48].

### 2.6. Telomerase 

Telomerase activity is usually detected in most cancer cells and is critical for cancer cell development [73]. However, deactivation of telomerase activity is one of the important strategies to inhibit cancer development. Natural products or bioactive ingredients of medicinal plants play a significant role in cancer prevention through the inhibition of telomerase activity. The expression level of the human telomerase reverse transcriptase gene (hTERT), as a catalytic subunit of the telomerase gene, was examined in kaempferol- and 5-Fluorouracil (5-FU)-treated cervical cancer (HeLa) cells. Kaempferol (100 mM) significantly decreased the gene expression of hTERT in combination with 5-FU and is a cytotoxic chemotherapy medication used to treat cancer (10 mM) 0.4, 0.3 and 0.3 times more effectively as compared to controls in the time durations of 24, 48 and 72 h, correspondingly [49].

### 2.7. Signal Transducer and Activator of Transcription 3 (STAT3) Pathway

Signal transducer and activator of transcription 3 (STAT3) is a cytoplasmic transcription factor as well as a member of the STAT protein family [74]. It is the most common and is over-expressed or constitutively stimulated in almost 75% of solid as well as hematological tumors [75]. Kaempferol deactivates as well as prevents nuclear localization of main transcription factors STAT3 and NF-κB, mutually accountable for cyclooxygenase-2 induction in response to IL-6. Furthermore, STAT3 and NF-κB were at the same time deactivated by kaempferol in acute inflammation [44]. A study based on ovarian cancer was made to evaluate the effects of kaempferol on the Mitogen-activated protein kinase (MEK) MEK/extracellular signal-regulated kinases (ERK) as well as STAT3 signaling pathway of ovarian cancer cells. Kaempferol showed a concentration-dependent reduction in the phosphorylation of p-MEK and p-ERK, whereas no clear effect has been seen on the expression of ERK and total MEK. Likewise, the phosphorylation of p-STAT3 (Ser 727) and p-STAT3 (Tyr 705) was strongly decreased, whereas total STAT3 remained unchanged [34]. 

### 2.8. Transcription Factor (AP-1) Pathway

Activator protein 1 (AP-1) is a transcription factor that regulates gene expression and it is identified to express cancer-relevant genes that activate pro-angiogenic, mitogenic and anti-apoptotic signals [76,77,78,79]. Activator Protein (AP-1) is linked with tumor progression and high levels of NF-κB and expression of AP-1 is seen in brain tumor gliomas [80]. Natural compounds show a role in the inhibition of cancer development through suppression of transcription factor AP-1. EGCG was described to suppress the AP-1 activation, cell transformation and inhibition of Ras-activated AP-1 [81,82]. A study based on osteosarcoma reported that kaempferol reduced the DNA binding activity of AP-1, an action probable to effect the decreased expression of matrix metalloproteinases (MMPs) (2, 9) and uPA. Together, this study demonstrated that kaempferol attenuated the Mitogen-activated protein kinases (MAPK) signaling pathways including ERK, JNK and p38 and resulted in the decreased DNA binding ability of AP-1, and henceforth, the reduction in the expression and enzymatic actions of MMPs (2, 9) as well as uPA, contributing to the inhibition of metastasis of osteosarcoma [35]. 

Human prostate cancer cells, firmly transfected with AP-1 luciferase reporter gene, were treated with flavonoids such as isoflavones, flavonols, flavonones and flavones. The maximum AP-1 luciferase induction of about three-fold over control was seen with 20 μM concentrations of genistein, quercetin, chrysin and 50 μM kaempferol. At higher concentrations, most of the flavonoids established prevention of AP-1 activity. Additionally, pretreating the cells with precise inhibitors of JNK decreased the AP-1 luciferase activity that was caused by genistein, whereas pretreatment with a MEK inhibitor decreased the AP-1 luciferase activity caused by kaempferol [45]. 

### 2.9. Nrf2 Pathway

Nuclear factor erythroid 2–related factor 2 (*Nrf2*) is a cytoprotective transcription factor that exhibits negative as well as positive effects on cancer [83,84]. The chemotherapy resistance role of Nrf2 has been noted by way of stimulating the metabolism of the drug and drug efflux [85]. Kaempferol plays a major role in cancer prevention through modulation of the Nrf2 signaling pathway. A study based on lung cancer reported that kaempferol precisely decreases Nrf2 mRNA and protein levels. A lower level of nuclear Nrf2 decreases the transcription of *Nrf2* target genes. Kaempferol (25 μM) facilitated the reduction in heme oxygenase 1 (HO-1), the initial as well as the rate-limiting enzyme, and Glutathione S-transferases (GST) phase II detoxification enzymes, and NQO1 (NAD(P)H Quinone Dehydrogenase 1) expression was noticed subsequently stimulating Nrf2 via tert-butylhydroquinone. Additionally, kaempferol incubation did not change the levels of pNFκBp65 as well as NFκBp65, signifying that it hinders the Nrf2 signaling pathway. Inhibition of Nrf2 via kaempferol causes the accumulation of reactive oxygen species (ROS) and makes lung cancer cells sensitive to apoptosis [50]. Kaempferol significantly increased the hepatic total and nuclear levels of the Nrf2 and superoxide dismutase (SOD) enzymes that catalyze the dismutation of superoxide radicals, heme oxygenase-1 and reduced glutathione (GSH) levels. To sum up, the protective effect of kaempferol against cadmium chloride-induced liver damage is mediated by anti-inflammatory effects and antioxidants motivated by suppressing the NF-κB p65 and keap1 and upregulating the Nrf2/HO-1 axis [86].

### 2.10. Phosphatidylinositol-3-Kinase (PI3K)/AKT Pathway

Phosphatidylinositol-3-kinase (PI3K)/AKT/mammalian target of rapamycin (mTOR) signaling is one of the vital intracellular pathways, which regulates cell growth, survival, motility, metabolism and angiogenesis [87,88]. The PI3K/AKT/mTOR pathway is one of the most frequently activated signaling pathways in cancers. Therefore, huge efforts have been made to find new drugs targeting PI3K/AKT signaling and downstream or upstream of the pathway [89,90,91]. Several natural compounds have shown a confirmed role in controlling PI3K/AKT pathways. Apigenin has been recognized to inhibit the PI3K/AKT/mTOR pathway, thus inducing apoptosis as well as autophagy in liver cancer cells [92]. To investigate whether AKT activation was observed in the synergistic effects of kaempferol and 5-Fluorouracil, the levels of PI3K, PTEN, p-AKT and AKT in colorectal cancer cells were examined. The p-AKT levels were reduced in cells treated with kaempferol and increased after 5-Fluorouracil treatment. Though, PI3K as well as p-AKT levels were substantially decreased in cells subjected to combination treatment when compared with 5-Fluorouracil alone. Thus, kaempferol as well as 5-Fluorouracil may promote a synergistically decreased colon cancer cell growth via inhibiting the PI3K/Akt pathway [51]. 

A study was performed to identify the sensitization effect of kaempferol on Erlotinib monotherapy in pancreatic cancer. In vitro results demonstrated that the combination of kaempferol and Erlotinib significantly promoted cell apoptosis and inhibited cell proliferation as compared to that with Erlotinib alone. Moreover, kaempferol sensitizing pancreatic cancer to Erlotinib treatment may probably be related to the PI3K/AKT signaling pathway and Epidermal growth factor receptor (EGFR), a transmembrane protein involved in TKI resistance. Furthermore, the combination of kaempferol and Erlotinib effectively downregulated the expression of Bcl-2, p-EGFR, p-AKT and p-ERK1/2 and upregulated the expression levels of cleaved PARP, a protein (enzyme) found in cells, Bax and cleaved caspase-9. These data suggest that kaempferol may be an effective therapeutic candidate to potentiate pancreatic cancer cell sensitivity to Erlotinib via the inhibition of EGFR and PI3K/AKT signaling [93]. Apoptosis was induced in cancer cells by kaempferol and it also induced aging via reduction of the hTERT as well as PI3K/AKT pathways [49].

### 2.11. ERK/p38 MAPK Pathway

p38 is a multitasking kinase that controls multiple cellular functions, such as stress response, cell proliferation, differentiation, cell migration and survival, apoptosis and also interacts with a plethora of substrates [94,95]. The p38 mitogen-activated protein kinases’ (p38 MAPKs) pathways are triggered in response to numerous cellular and environmental stresses, inflammation and other signals [96,97]. Increased expression as well as activity of p38 MAPK correlates with poor prognosis in several types of cancers [98,99], and thus the role of p38 MAPK inhibitors has become increasingly significant to be used in cancer therapeutics [100]. In this regard, natural compounds such as curcumin showed phosphorylation of c-Jun N-terminal kinase (JNK) and p38 mitogen-activated protein kinase (MAPK). Additionally, JNK and p38 MAPK inhibitors meaningfully suppressed curcumin-induced activation of caspases-9/-3 and inhibited the apoptosis of Y79 cells. Overall, findings have advocated that curcumin induced the apoptosis of retinoblastoma Y79 cells via the JNK and p38 MAPK pathways’ activation [101]. 

Similarly, a recent study reported that with the activation of VEGFR2, HIF-1α, which is a subunit of a heterodimeric transcription factor hypoxia-inducible factor 1, was suppressed by kaempferol and it also suppressed other markers of the ERK/p38 MAPK and PI3K/Akt/mTOR signaling pathways in endothelial cells. These outcomes recommend that kaempferol prevents angiogenesis via decreasing VEGFR2 and HIF-1α activation through ERK/p38 MAPK and PI3K/Akt/mTOR signaling in endothelial cells [36]. Colorectal cancer-based findings demonstrated that kaempferol increased the sub-G1 population and G2/M arrest in HCT116 colorectal cancer cells. Similarly, kaempferol increased the PARP cleavages and activation of caspase-8, -9 and -3; phospho-p38 MAPK; p21; and p53 in HCT15 and HCT116 cells (human colorectal carcinoma cell line) [46]. 

Kaempferol enhanced apoptosis and radically upregulated DR5, ERK1/2, CHOP, DR4, JNK, apoptotic protein and the expression of p38 and also caused a decline in the expression of anti-apoptotic proteins. Finally, kaempferol sensitized ovarian cancer cells to TNF-related apoptosis-inducing ligand (TRIAL)-induced apoptosis through upregulation of DR4 and DR5 via the ERK/JNK/CHOP pathways [52]. Kaempferol inhibited the protein phosphorylation of ERK, p38 and JNK; however, the inhibition was in different kinetics for all three kinases. Important inhibition was seen for ERK as well as p38 after 4 h of treatment with the drug, though 6 h of drug treatment was essential to prevent the protein phosphorylation of JNK. Overall, the findings showed that kaempferol inhibits protein phosphorylation and, henceforth, the inactivation of ERK, JNK and p38, influential to the reduction in the expression and activities of MMP [35].

### 2.12. Wnt/β-Catenin Signaling Pathway

The Wnt/β-catenin signaling pathway, also known as the canonical Wnt signaling pathway, is a conserved signaling axis contributing to apoptosis, proliferation, differentiation, invasion, migration and tissue homeostasis [54,102,103]. WNT/β-catenin signaling controls the self-renewal as well the migration of CSCs, thus promoting tumor growth and metastasis in breast [104] cancer. Kaempferol, a flavonoid, shows a role in cancer inhibition through regulation of the Wnt/β-catenin signaling pathway. Kaempferol causes osteoblastic differentiation through the activation of the Wnt signaling pathway. The stimulation of SaOS-2 cell osteoblasts via kaempferol caused an increased activity of the Wnt-responsive reporter construct, Axin-2, and, consequently, stabilized Wnt signaling facilitated transcription factor β-catenin, which is important for the activation of Wnt-targeted genes for osteogenesis. The kaempferol-caused ALP activity was completely abolished by FH 535, an inhibitor of the Wnt signaling pathway [105]. A recent research study based on retinoblastoma reported that QRT-PCR and Western blot showed that co-transfection with pcDNA-PGC1α and pCMX-ERRα/plasmids in SO-RB50 cells significantly increased the mRNA as well as protein expression levels of cyclin D1, β-catenin and c-myc and decreased the mRNA and protein expression levels of GSK3β. Kaempferol treatment meaningfully suppressed the mRNA and protein expression levels of c-myc, β-catenin and cyclin D1 and increased the mRNA and protein expression levels of GSK3β [53].

### 2.13. Cell Cycle 

Checkpoints of the cell cycle are important in monitoring as well as regulating the cell cycle progression [106,107,108,109,110]. Cell cycle alterations initiate the growth of cancer [111,112]. Moreover, initiation of cell cycle arrest at several checkpoints of the cell cycle has antitumor potential [113]. Bioactive ingredients of herbal origin play an important role in cancer management through cell cycle arrest. Kaempferol treatment suppressed MDA-MB-231(epithelial human breast cancer cell line) cell proliferation, and it was examined whether it happened via the induction of cell cycle arrest. The data showed that kaempferol decreased the population of cells in the G_1_ phase, and the population of cells in the G2 phase was consequently enhanced, which indicated that kaempferol contributed to the G2/M arrest induction. These outcomes demonstrated that kaempferol endorsed the inhibition of MDA-MB-231 cells through the induction of cell cycle arrest [47] and kaempferol-induced growth inhibition is dominated by cell cycle changes [114].

The role of kaempferol in cell cycle arrest was also investigated in renal cell carcinoma. Kaempferol treatment (100 mM) for 24 h led to cells arrested mostly at the phase G2-M stage with 43.45% in 769-P cells and 52.36% in 786-O cells. Similarly, it was noticed that after treatment with different doses of kaempferol, many cell cycle-related gene expressions were altered. For example, the levels of p21waf1/Cip1 and Chk1 were enhanced, while the levels of p35, Cyclin-Dependent Kinase 2 (CDK2) and cyclin B1 were decreased in cancer cells [37]. Similarly, different doses of kaempferol were used to treat the SK-HEP-1 cells and to investigate its effect on cell cycle arrest. The sub-G_1_, G_1_ and G2 phases were different between the kaempferol-treated and control groups. Treatment by kaempferol caused G2/M cell cycle arrest, and the expression of cyclin B and *CDK1* decreased in kaempferol-treated SK-HEP-1 cells [29].

## 3. Kaempferol: Role in Management of Various Cancers

**Figure 3:**
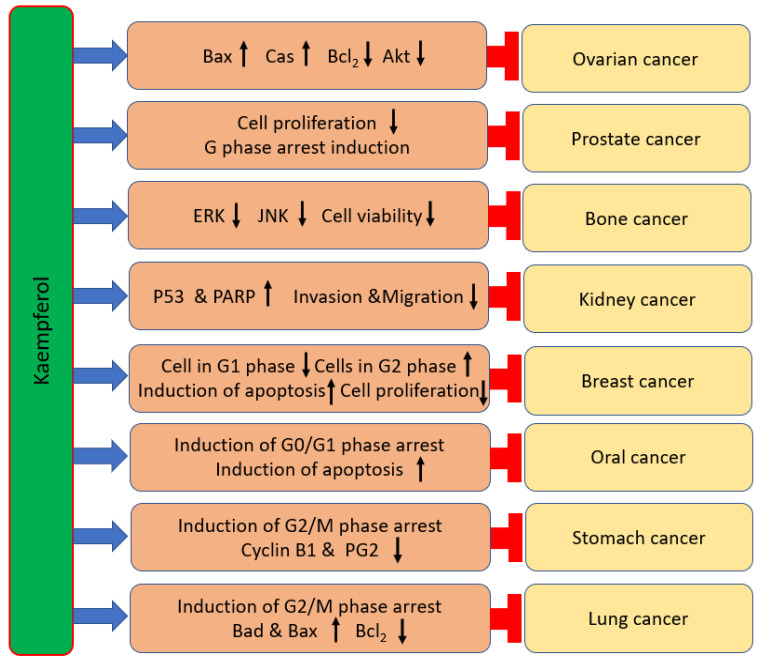
Role of kaempferol in the inhibition and treatment of cancers through induction of apoptosis [34,47,117], cell cycle arrest [118,119,120], activation of tumor suppressor gene [37] and inhibition of invasion and migration [121]. Up-arrow (↑) indicates up-regulation and down-arrow (↓) indicates down-regulation.

Cancer is the leading cause of death worldwide and approximately 18.1 million new cases of cancer and 9.6 million deaths occurred worldwide in 2018, and the new cases will be approximately 20 million worldwide by 2025 [2,3]. Cancer is a multifactorial disease leading to cellular differentiation and loss of growth control that ultimately leads to tumor development [115]. Natural compounds have confirmed their role in cancer prevention through modulation of various cell signaling pathways [116]. In this regard, the role of kaempferol in various cancers is described below (Table 2 and Figure 3).

### 3.1. Cervical Cancer

At the macro level, a 3-(4,5-dimethylthiazol-2-yl)-2,5-diphenyl-2H-tetrazolium bromide (MTT) assay showed that kaempferol plays a role in the inhibition of cervical cancer cell proliferation. Furthermore, it was observed that kaempferol induced apoptosis, intracellular free calcium elevation and mitochondrial membrane potential disruption. At the micro level, kaempferol abolishes the networks of microtubule formation. Moreover, the spindle shape of cancer cells is shortened after treatment with kaempferol, with significantly increased roughness of the cell surface [146]. Cell viability was decreased by kaempferol treatment in a concentration- and time-dependent manner. Similarly, kaempferol induced cellular apoptosis as well as aging via the PI3K/Akt and hTERT pathways through its downregulation. This study proposes that this compound may be a valuable adjuvant beneficial agent for cervical cancer treatment [49]. After the treatment of cervical cancer (HeLa cells) with various concentrations of kaempferol, it was described that kaempferol decreases cell viability. Furthermore, kaempferol (30 to 50 µM) changed the appearance of treated cancer cells in comparison to the control and also induced cell death. In addition, the cell cycle at the G2/M phase was halted after kaempferol treatment; the cells’ population was higher in a dose-dependent way as compared to the Dimethyl sulfoxide (DMSO), which is an organosulfur compound used as the control [58].

### 3.2. Breast Cancer

In women, breast cancers are the most frequently diagnosed cancer and are the foremost cause of death among the cancer worldwide [147]. The current line of treatment for breast cancer is expensive and also causes adverse effects on health. In this vista, kaempferol plays an important role in the prevention of breast cancer. The antiproliferative effects of kaempferol in triclosan-caused cell growth were observed in breast cancer cells. Results demonstrated that triclosan promoted the cell viability of MCF-7 (breast cancer cells) through estrogen receptor α. Instead, kaempferol pointedly suppressed E2 or triclosan-caused cell growth. In an in vivo xenografted mouse model, tumor growth was prompted by treatment with E2 or triclosan. E2- or triclosan-induced breast tumor growth was inhibited by co-treatment with kaempferol, which is consistent with results of in vitro studies [148].

To examine the potential role of E2 or TCS as well as each combination with kaempferol in cancer proliferation in an in vivo model, the xenografted mice with MCF-7 breast cancer cells were examined in the animal experiment scheduled. As a result of administration of E2, TCS or kaempferol for 6 weeks, the tumor volumes of the E2 and TCS groups were constantly increased compared to that of the vehicle. On the other side, the tumor volumes of the combination groups of kaempferol with E2 were meaningfully decreased compared to that of a single treatment group of E2. Although there was no statistical significance, kaempferol showed the tendency to prevent the tumor growth effect of TCS. The tissue sections of tumors from the mice exposed to E2 or TCS showed a hyperproliferative cellular formation with greater density compared to corn oil, whereas those from the mice treated with the combination of TCS and kaempferol or E2 and kaempferol exhibited more disassembled or sparse cellular structures compared to a single treatment of E2 or TCS [148].

It was examined whether kaempferol induced the expressions of DNA damage-associated proteins and apoptotic proteins. The outcomes presented that kaempferol increased the expression levels of cleaved caspase 3, cleaved caspase 9 and p-ATM as compared to the control sample. Remarkably, the suppressive role of kaempferol on cell proliferation was high in MDA-MB-231 cells as compared to the estrogen receptor-positive BT474 cell line. Additionally, after kaempferol treatment for 48 h, the population of cells in the G_1_ phase was substantially decreased, and the population of cells in the G2 phase increased evidently, indicating that kaempferol contributed to the induction of G2/M arrest [47]. The low dose of kaempferol (10–40 μmol/L) significantly inhibited the migration of MDA-MB-231 and MDA-MB-453 cells. Additionally, the low dose of kaempferol (20 and 40 μmol/L) did not inhibit the migration of SK-BR-3 HER2^+^ breast cancer cells and MCF-7 ER^+^/PR^+^ breast cancer cells. The activations of Rac1 and RhoA were meaningfully blocked by treatment of kaempferol in MDA-MB-231 and MDA-MB-453 cells [122]. 

Another study based on breast cancer reported that kaempferol (100 µM) showed antiproliferative and cytotoxic properties, which were mimicked by low extracellular glucose conditions as well as reversed by high extracellular glucose conditions. Lastly, treatment of cells with kaempferol brought an increase in extracellular lactate levels over time, due to the prevention of MCT1-mediated lactate cellular uptake. The cytotoxic and antiproliferative potential of kaempferol in these tested cells seems to be dependent on this mechanism [123]. An important study reported that kaempferol treatment was confirmed to have an important role in the reduction of cell viability, an effect which occurs in a dose-dependent way [124].

### 3.3. Ovarian Cancer

An important study was performed to evaluate whether kaempferol inhibits ovarian cancer by activation of endoplasmic reticulum stress and autophagy, and to establish its influence on the sensitivity of ovarian cancer cells to cisplatin. Results demonstrated that compared to control cells, kaempferol decreased viability by 53.17%, increased cell apoptosis by 158% and decreased proliferation by 49.17% in A2780 ovarian cancer cells. In parallel, it increased protein levels of PERK, GRP78, IRE-1, ATF6, LC3II, beclin 1 and caspase 4, thus proposing activation of cytotoxic autophagy. This was facilitated by increasing intracellular Ca^+2^ levels. Additionally, kaempferol increased the sensitivity of ovarian cancer A2780 cells to cisplatin via decreasing the p-Akt protein levels [125]. To establish whether kaempferol stimulates apoptosis in the ovarian cancer cells, AO/EB staining was accomplished which presented notable changes in the membrane blebbing and nuclear morphology of the cancer cells. In addition, DAPI staining also displayed an increased number of white-color nuclei suggestive of apoptosis. Similarly, the treatment of OVACAR-3 cells (cervical cancer cell lines) with 50 µM kaempferol significantly increased the apoptotic cell percentage from 3.46% in the control to 34.16%. The apoptosis was additionally confirmed by the increased expression of Bax, and Caspase 3, 8, 9 and Bcl-2 expression decreased in cancer cells [34]. Further, kaempferol nanoparticles showed strong inhibition of ovarian cancer cell viability [149].

Kaempferol increased apoptosis and upregulated ERK1/2, p38, DR4, DR5, JNK, CHOP and apoptotic protein expression and decreased the expression of anti-apoptotic proteins [52]. Treatment of ovarian cancer A2780/CP70 cells with kaempferol increased the activities of caspase 3/7 significantly. Similarly, elevated levels of caspase 3/7 were noticed in three ovarian cancer cell lines treated with kaempferol (80 μM) and a dose-dependent response was observed (0 to 80 μM) in all three ovarian cancer cells [117].

### 3.4. Endometrial Cancer

In endometrial cancer cells, the antitumor potential of kaempferol was investigated. The finding described that kaempferol effectively suppressed viability of two ER-positive endometrial cancer cell lines. Moreover, kaempferol induced apoptotic cell death and sub-G1 cell accumulation in a dose-dependent way. Further studies show that kaempferol showed apoptotic cell death largely via suppressing *Bcl*-*2* (B-cell lymphoma 2), Erα, types of estrogen receptors and survivin protein. Consequently, the outcomes of the existing study recommended that targeting survivin and ERα with kaempferol might be a unique therapeutic choice against endometrial carcinoma [126]. Kaempferol showed substantial and selective anti-cancer effects on endometrial carcinoma cells (MFE-280). The anti-cancer effects were established to be due to G2/M phase cell cycle arrest and activation of the mitochondrial-mediated apoptotic pathway. In addition, the kaempferol expressively inhibited cell migration as well as cell invasion properties of these cancer cells. Moreover, as compared to the untreated cells, kaempferol-treated cells showed a dose-dependent downregulation of p-PI3K, p-mTOR and p-Akt proteins [127]. 

### 3.5. Prostate Cancer

Kaempferol effectively suppressed androgen-independent and -dependent prostate cancer cells’ proliferation and plays role in the induction of apoptosis. Additionally, it was noticed that kaempferol brought the cell cycle to be arrested at the G_1_ phase in 22Rv1 cells, while in PC-3, classical prostate cancer cell line cells, this occurred at the S and G2 phases. Furthermore, it was observed that mRNA as well as the protein of Ki67 (proliferation marker), which is responsible for the cell proliferation in prostate cancer, were substantially inhibited by kaempferol treatment in both androgen-dependent and androgen-independent prostate cancer cells. Overall, kaempferol prevented the androgen-dependent and -independent prostate cancer cells’ proliferation via controlling the Ki67 expression [150]. 

Again, the role of kaempferol in the apoptosis of prostate cancer cells was investigated. Results confirmed that kaempferol could promote apoptosis of LNCaP (androgen-sensitive human prostate adenocarcinoma cells), AR-positive prostate cancer cells, in a dose-dependent manner. It is worth observing that the role of kaempferol in the apoptosis of prostate cancer cells is in a dihydrotestosterone (DHT)-dependent manner. Moreover, kaempferol presented the capacity to suppress AR transactivation caused by DHT (1 nM), and this inhibition potential of kaempferol was dose-dependent. AR target gene (PSA, TMEPA1 and TMPRSS2) mRNA levels were enhanced by DHT. Then, kaempferol in concentrations such as 5 μM, 10 μM and 15 μM efficiently suppressed the DHT-induced AR target gene expression in LNCaP cells [128]. 

### 3.6. Bladder Cancer

Another study based on nude mice bearing bladder cancer was performed to evaluate the role of kaempferol in cancer. The genomic DNA were extracted from xenografts and the methylation changes were examined. The findings demonstrated that kaempferol modulated DNA methylation in bladder cancer. Further, kaempferol precisely inhibited the protein levels of DNMT3B without changing the expression of DNMT1 or DNMT3A. These results proposed that kaempferol could induce the degradation of DNMT3B via the ubiquitin-proteasome pathway [151].

Treatment of bladder cancer cells with different concentrations of kaempferol enhanced the expression levels of p-p53 and p-BRCA-1, whereas the expression level of total p53 marginally decreased as compared to the negative control. In the meantime, kaempferol enhanced the pro-apoptotic proteins and downregulated the expression of anti-apoptotic proteins in bladder cancer EJ cells. The SubG_1_ phase as well as the S phase of cancer cells effectively increased when bladder cancer cells were treated with an increasing concentration of kaempferol [129]. An important study based on bladder cancer was performed in which levels of PTEN and Akt in EJ cells were observed on treatment with kaempferol. It was noticed that expression of PTEN increased in a time-dependent fashion in EJ cells treated with 40 μM kaempferol, while Ser473-phosphorylated Akt (pAkt) expression was significantly decreased after treatment with 40 μM kaempferol. These results designated that kaempferol upregulates PTEN expression, whereas it inhibits pAkt (Ser473) in EJ cells [33]. Kaempferol showed increased bladder cancer cell cytotoxicity and induced apoptosis as well as cell cycle arrest. Consistent with the in vitro finding, it was detected that kaempferol-treated mice had substantially suppressed tumor growth. Furthermore, in vitro as well as in vivo results presented that the inhibition of bladder cancer invasion as well as metastasis was achieved by kaempferol treatment [152]. 

The bladder cancer xenograft mouse model was developed by administrating 5637 bladder cells subcutaneously into the nude mice. At the end of treatment, all mice were sacrificed and tumor weights in each experimental group mice were measured. Amazingly, substantial differences in tumor weights between the control group and kaempferol-treated group mice were noticed. Mice from all three test groups exhibited a decrease in tumor weights and the reduction was more noteworthy with the high-dose drug treatment, demonstrating the positive effect of this compound in efficiently decreasing tumor growth. These results proposed the effective in vivo antitumor efficacy of kaempferol in treated doses against human bladder tumors without host toxicity, and in vitro data exhibited low toxicity to normal human uroepithelial cell-SVHUC. Further, the TUNEL assay showed that apoptotic cells increased from 7% in control group mice to 70% in the 150 mg/kg kaempferol-treated mice [152]. 

### 3.7. Renal Cell Carcinoma

The effect of kaempferol on apoptosis in renal cancer cells was evaluated. The flow cytometry finding revealed that Annexin V-positive cells increased after kaempferol treatment. Additionally, p53 as well as cleaved PARP increased after treatment with kaempferol [37]. Kaempferol also decreased the invasion as well as the migration of RCC 786-O cells. The outcomes from the wound healing assay demonstrated that kaempferol expressively decreased cell migration dose-dependently in 786-O cells [121].

### 3.8. Liver Cancer

SK-HEP-1 human hepatic cancer cells were treated with different doses of kaempferol in order to observe whether it arrests the cell growth in a particular cell cycle phase. The sub-G1, G1 and G2 phases were meaningfully different between the kaempferol-treated and control groups. Kaempferol treatment showed a role in the G2/M cell cycle arrest, and the expression of cyclin B and CDK1 decreased in kaempferol-treated cancer cells. Moreover, after kaempferol treatment, acidic vesicular organelle (AVO)-positive cells increased in a concentration-dependent way in comparison with the control. Based on this outcome, it was concluded that kaempferol induced autophagy [29]. Kaempferol treatment of Huh7 cells under hypoxic conditions efficiently inhibited hypoxia-inducible factor 1 (HIF-1) activity in a dose-dependent manner. Kaempferol treatment of Huh7 cells caused an important reduction in viability, which was significantly more obvious in the hypoxic conditions [130].

### 3.9. Gastric Cancer

Kaempferol led to a noticeable decrease in the protein levels of cyclin B1, Cdc25C and Cdk1 in a dose-dependent manner in gastric cancer cells. These findings demonstrated that the inhibitory effect of kaempferol was related to the induction of G2/M phase arrest. Moreover, an in vitro study showed that kaempferol treatment pointedly decreased the expression levels of p-ERK, cyclooxygenase-2 (COX-2) and p-AKT, which participated in cell cycle arrest and cell proliferation [118]. Another study was performed to investigate the biological potential and molecular mechanism taking part in the kaempferol-initiated treatment of gastric cancer. Kaempferol treatment showed enhanced autophagy, cell death, downregulation of p62 and increased conversion of LC3-I to LC3-II. In addition, this finding revealed that kaempferol induces autophagic cell death through the activation of the IRE1-JNK-CHOP signaling pathway, representative of the ER stress response. Additionally, kaempferol facilitated epigenetic changes through the inhibition of G9a (HDAC/G9a axis) and triggered autophagic cell death [48].

### 3.10. Pancreatic Cancer

Kaempferol efficiently inhibited the migratory activity of human pancreatic cancer cells. The anti-cancer potential of kaempferol was facilitated by the blockage of ERK1/2, EGFR-related Src and AKT pathways. To examine the anti-cancer effect of kaempferol and its derivatives, various concentrations of kaempferol, kaempferol-3-O-glucoside and kaempferol-4′-O-glucoside were used to treat pancreatic cancer cells. Results confirmed that kaempferol (50 μM) precisely inhibited the cell viability of cancer cells, whereas kaempferol-4′-O-glucoside and kaempferol-3-O glucoside did not have any inhibitory effect on cancer cells [131]. A recent study was made to examine whether kaempferol efficiently suppresses pancreatic cancer via upregulation of ROS, and to discover the causal molecular mechanism. The findings revealed that kaempferol efficiently suppressed pancreatic cancer based on in vivo and in vitro studies. In addition, kaempferol endorsed apoptosis in vitro by increasing ROS generation, which was involved in Akt/mTOR signaling. Decreased TGM2 mRNA and protein levels were noticed in the cells after treatment with kaempferol [67].

### 3.11. Bile Duct Cancer

Bile duct cancer (BDC) has been recognized as a highly aggressive cancer arising from epithelial cells of the bile duct, including intrahepatic, perihilar and extrahepatic varieties [153]. A study based on cholangiocarcinoma reported that kaempferol meaningfully suppressed the viability of cholangiocarcinoma cells in a time- and dose-dependent way and inhibited the proliferation of cholangiocarcinoma cells in a dose-dependent fashion. Furthermore, kaempferol reduced the number of colonies in a dose-dependent manner and induced apoptosis. Kaempferol meaningfully decreased the migration and reduced the invasion ability of cholangiocarcinoma cells (CCA) in a dose-dependent way. Kaempferol significantly inhibited the growth of the tumor as compared to the control group. Additionally, kaempferol was established to reduce the average volume and number of foci per mouse, as compared with the control [132].

### 3.12. Gall Bladder Cancer

SGC996 and GBC-SD cells were used to evaluate the effect of kaempferol, and it was reported that it was significantly arrested in the G_0_/G_1_ phase and showed an important decrease in the S and G2/M phases in a dose-dependent fashion with the increase in the concentration of kaempferol. For the meantime, kaempferol meaningfully induced cell apoptosis of both cancer cells, SGC996 and GBC-SD cells, in a dose-dependent way. These outcomes designated that kaempferol potentially endorses apoptosis of gall bladder cancer cell lines partly via arresting gall bladder cancer cells at the G_0_/G_1_ phases. Moreover, kaempferol meaningfully prevented tumor progression, tumor weight and volume, which were suggestively decreased by kaempferol compared with that in the control group. These results recommend that kaempferol could prevent gall bladder cancer progression in vivo [133].

### 3.13. Oral Cancer

Kaempferol considerably inhibited clone formation and tumor cell proliferation in vitro in oral cancer cells. After kaempferol treatment, tumor cells were induced to G_0_/G_1_ phase arrest, and the expression of protein involved in the regulation of the cell cycle was changed. Furthermore, kaempferol showed a direct effect on EGF receptor activity and the downstream signaling pathways from this receptor were clearly decreased. The antitumor potential of kaempferol was confirmed in xenograft model and kaempferol noticeably controlled tumor growth in vivo. In the meantime, intense decreases in epidermal growth factor receptor activity as well as hexokinase-2 expression were detected in kaempferol-treated tumor tissue [119]. Kaempferol suggestively inhibited human esophageal squamous carcinoma Eca-109 cells’ proliferation in a concentration-dependent fashion and induced clear cell apoptosis. In addition, kaempferol treatment caused upregulated Bax as well as downregulated Bcl-2 mRNA expression. A further increase in caspase-3/9 activities was observed in human esophageal squamous carcinoma Eca-109 cells following kaempferol treatment [154]. Kaempferol considerably inhibited the migration and invasion of SCC4 cells in a dose-dependent manner. Additionally, MMP-2 (member of the *MMP* gene family) promoter activity was decreased in a dose-dependent fashion, demonstrating that kaempferol inhibits expression of MMP-2 at the transcriptional level [134].

### 3.14. Lung Cancer

The in vitro and in vivo study was performed to evaluate the role of kaempferol in lung cancer. Kaempferol treatment inhibited the growth of lung cancer cells via activation of the apoptotic pathway. Moreover, this compound induced G2/M cell cycle arrest as well as the increase in radiation-triggered death. The in vivo result exhibited that kaempferol increased tumor cell apoptosis. To sum up, the results confirmed that kaempferol increased tumor cell killing via radiation under in vitro conditions. Additionally, inhibition of the ERK and Akt/PI3K pathways and triggering of the mitochondria apoptotic pathway was noticed in the in vivo findings [120]. Kaempferol-initiated H460 cell apoptosis is a distinctive apoptosis that is conveyed by an important condensation of DNA and an enhancement of intracellular Adenosine triphosphate (ATP) (source of energy for use and storage at the cellular level). Kaempferol-caused apoptosis is linked to its capability to change the expression of apoptotic markers [155]. In lung cancer A549 cells, kaempferol treatment caused a decrease in cell viability in a dose- and time-dependent manner. Alongside, kaempferol treatments led to an increase in Bax and Bad, whereas the expression of Bcl-x_L_ and Bcl-2 was inhibited in a dose-dependent way and the MAPK was activated, and Akt-1 and phosphorylated Akt-1 were also inhibited upon kaempferol treatment [156]. Akt1 was needed for the TGF-β1-initiated induction of the epithelial-to-mesenchymal transition and cell migration, and kaempferol entirely abolished the TGF-β1-caused Akt1 phosphorylation. Overall, kaempferol blocks the TGF-initiated epithelial-to-mesenchymal transition as well as the migration of lung cancer cells [157].

### 3.15. Bone Cancer

The anti-metastatic potential of kaempferol and its molecular mechanism of action in human osteosarcoma cells was investigated. Inhibitory effects on the invasion and adhesion of the osteosarcoma were noticed after kaempferol treatment in a concentration-dependent manner. Additionally, kaempferol inhibited the migration of osteosarcoma cancer cells in a concentration-dependent way at different treatment time points. Further experiments exhibited that kaempferol treatment decreases the enzymatic activities and protein levels of MMP 2,9 and urokinase plasminogen activator. Moreover, kaempferol was capable of decreasing the protein phosphorylation of ERK, JNK and p38. It was also reported that kaempferol decreased the AP-1DNA binding activity, an action possible to consequence in the decreased expression of MMP-2,-9 and uPA [102]. Kaempferol significantly decreases the cell viabilities of HOB, U-2 osteosarcoma and 143B cells, particularly U-2 osteosarcoma cells, in a dose-dependent way. Additionally, kaempferol treatment impacted the time-dependent protein expressions and participated in the endoplasmic reticulum stress pathway as well as the mitochondrial signaling pathway. Moreover, pretreating cells via caspase inhibitors, BAPTA or calpeptin prior exposure to kaempferol enhances cell viabilities. The anti-cancer effects of kaempferol were examined based on an in vivo study in BALB/c (nu/nu) mice injected with U-2 osteosarcoma cells, and the outcomes designate tumor growth inhibition [135]. 

The effects of kaempferol on antiproliferative activity in BALB/cnu/nu mice after injection with human osteosarcoma U-2 OS cells was examined. Three groups of mice were individually treated with a DMSO control vehicle, 25 mg/kg of kaempferol or 50 mg/kg of kaempferol. Results showed that kaempferol meaningfully decreased the tumor weight as well as the tumor volume compared to the control group [135].

### 3.16. Colon Cancer

Kaempferol increased DNA fragmentation, chromatin condensation and the number of early apoptotic cells in a dose-dependent fashion. Additionally, kaempferol enhanced the levels of cleaved caspase-9, caspase-3 and caspase-7. Furthermore, it increased cytosolic cytochrome c concentrations and mitochondrial membrane permeability, and kaempferol increased those of Bik and decreased the levels of Bcl-xL proteins [136]. The activation of phosphorylation of Stat3 and Erk was decreased by kaempferol. An inhibitor of Stat3 phosphorylation encouraged morphological changes in KNC, colon cancer-type cells like those in kaempferol-treated cells, signifying that kaempferol-induced differentiation might be arbitrated by means of inhibition of Stat3 phosphorylation [137]. The combined treatment with kaempferol and tumor necrosis factor-related apoptosis-inducing ligand (TRAIL) radically induced apoptosis in human colon cancer SW480 cells as compared to TRAIL treatment alone. Kaempferol evidently upregulated TRAIL receptors, DR5 and DR4 [138]. 

### 3.17. Esophagus Cancer

Esophageal cancer (EC) is the ninth most common cancer and the sixth leading cause of cancer deaths worldwide [1]. A study was performed to examine the role of kaempferol on esophageal cancer and its mechanisms of action as an anti-cancer agent. Results exhibited that kaempferol significantly inhibited clone formation and tumor cell proliferation in vitro. Moreover, tumor cells were brought to G0/G1 phase arrest after treatment of kaempferol and the expression of proteins that participated in cell cycle control was intensely changed. Finally, the antitumor activity of kaempferol was authenticated in xenograft models and kaempferol obviously controlled tumor growth. In the meantime, a significant decrease of epidermal growth factor receptor activity and hexokinase-2 expression were seen in kaempferol-treated tumor tissue [119]. Kaempferol meaningfully inhibited Eca-109 cell proliferation in a concentration-dependent way and induced noticeable cell apoptosis. Moreover, after kaempferol treatment, the cells showed downregulated Bcl-2 and upregulated Bax mRNA expression. Additionally, there was a substantial increase in caspase-3 and caspase-9 activities in Eca-109 cells subsequent to kaempferol treatment [154].

### 3.18. Skin Cancer

Melanoma is a highly heterogeneous tumor, comprising various genetic and molecular alterations [158]. A recent study based on melanoma was performed and it was reported that kaempferol displays a noteworthy anti-cancer activity against melanoma cancer A375 cells. Moreover, the anti-cancer effect of kaempferol was recognized to be due to the initiation of apoptosis in melanoma A375 cells. Additionally, kaempferol showed the potential to initiate G2/M cell cycle arrest and inhibited the cell migratory effect of cancer cells [139]. The role of kaempferol on melanoma was investigated based on in vitro and in vivo experiments. The in vitro experiment reported that kaempferol clearly induced cell cycle arrest and cell apoptosis and inhibited cell viability of melanoma B16 cells. Moreover, in vivo results revealed that growth of mice xenografts was efficiently inhibited by kaempferol. More notably, kaempferol upregulated the number of NKT cells and CD8^+^ T cells and downregulated the number of MDSC cells [140]. 

Another study reported that kaempferol relates to RSK2 as well as MSK1 at the ATP-binding pocket and inhibits their corresponding kinase activities [141]. Kaempferol treatment showed a role in the inhibition of the migration and invasion of A375 as well as B16F10 cells and decreased the lung metastasis of melanoma cells. In conclusion, kaempferol inhibited melanoma metastasis through stopping the aerobic glycolysis of melanoma cells, in which the binding of HK2 as well as VDAC1 on mitochondria was broken [159]. 

### 3.19. Leukemia

Kaempferol and quercetin at a concentration of 40 µM reversed the proliferation of acute myeloid leukemia THP-1 cells in comparison with control cells. It demonstrated the synergistic effects of the kaempferol plus quercetin on THP-1 cell proliferation. Moreover, these compounds enhance Bax, caspase 3, and caspase 8 expressions, and a reduction in Bcl-2, Mcl-1 and Bcl-xl expression at mRNA levels in the treated cells was observed as compared to control cells [142]. Exposure of J/Neo cells to kaempferol showed the activation of the ATM/ATR-Chk1/Chk2 pathway, cytotoxicity, triggering of the phosphorylation of p53 (Ser-15), inactivation of cyclin-dependent kinase 1 (Cdk1), inhibitory phosphorylation of Cdc25C (Ser-216) and the resulting G2 arrest of the cell cycle. Under these conditions, mitochondrial membrane potential (Δψm) loss, upregulation of Bak and PUMA levels, activation of caspase-9, -8 and -3 and accumulation of apoptotic sub-G1 cells were induced [143]. Kaempferol + TRAIL treatment initiated apoptosis strongly in MOLT-4 cells at different durations (12, 24 and 48 h). Moreover, it was noticed that kaempferol could inhibit the expression of VEGF-β, X-IAP, c-FLIP, cIAP1/2 and FGF-8 and equally enhance the expression of DR4/5 in MOLT-4 cells. It was proposed that co-treatment of MOLT-4 cells with TRAIL + kaempferol is a practical and striking approach to eliminate cancer resistance to TRAIL via intracellular anti-apoptotic protein inhibition, upregulation of DR4/5 and by suppression of the FGF-8 and VEGF-β expressions [144].

### 3.20. Fibrosarcoma

An experiment was set up to evaluate the role of kaempferol in fibrosarcoma cells. The study reported that galangin and kaempferol effectively decreased MMP-9 secretion and decreased transcription of MMP-9 mRNA. Furthermore, galangin as well as kaempferol powerfully decreased the IκBα phosphorylation and considerably decreased the JNK phosphorylation in fibrosarcoma cells [145].

## 4. Bioavailability and Approaches to Improve the Efficacy 

Kaempferol is a polyphenol playing a major role in health management due to its antioxidant, anti-inflammatory, hepato-protective and anti-cancer properties through the modulation of various biological pathways. In order to investigate the health-beneficial role of kaempferol, it is important to know in detail about its pharmacokinetics and bioavailability. In spite of its implication in health management, the potential role of kaempferol in cancer management is still to be explored due to its poor bioavailability. The drug solubility, solution formation and its gastrointestinal permeability are indispensable aspects that regulate the level and speed of absorption along with the drug bioavailability [160]. Bioactive food compounds including polyphenols are comparatively poorly absorbed, with an absorption range from 0.3% to 43%, and the plasma concentrations of their metabolites can be lower [161]. However, low bioavailability hampers the use of bioactive food compounds as functional constituents [162,163]. A study was made to evaluate the kaempferol digestion and absorption from black tea. A total number of 15 participants were included who took 27 mg of kaempferol from black tea for three days. Kaempferol urinary excretion was 2.5% of the ingested amount and this result demonstrated that kaempferol absorption was more than quercetin [164]. 

Kaempferol was administered to male Sprague Dawley rats intravenously (i.v.) (10, 25 mg/kg) or by oral route (100, 250 mg/kg). After the i.v. method, the plasma concentration time–profiles for 10 and 25 mg/kg were consistent with high clearance (around 3 L/h/kg) and large volumes of distribution (8 to 12 L/kg). After oral administration, the plasma concentration–time profiles demonstrated a fairly rapid decline and the bioavailability was poor at around 2% [165]. Low absorption, low solubility, poor bioavailability and fast elimination and metabolism hindered its actual role in health management. 

**Figure 4:**
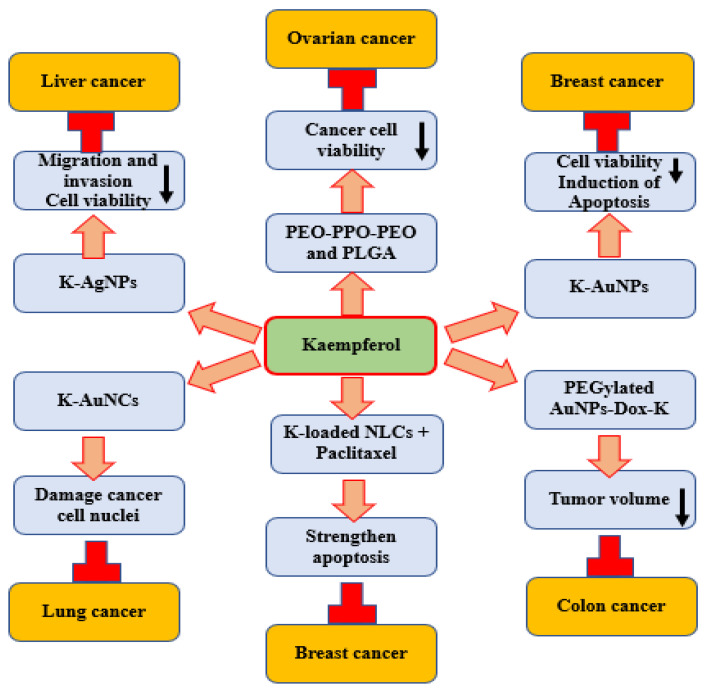
Kaempferol-based nanoformulations showed a significant effect on cancer cells’ inhibition through reduction in cell viability [149,166,167], induction of apoptosis [168], damage to cancer cell nuclei [167] and increase in LDH leakage percentage [169]. Down-arrow (↓) indicates down-regulation.

Nanotechnology-based approaches were used to enhance the bioavailability and disease management properties of kaempferol (Table 3) and (Figure 4). Nanoparticles incorporated with kaempferol were prepared and their effectiveness was evaluated in the inhibition of viability of cancerous as well as normal ovarian cells. A significant decrease in cell viability of both cancerous and normal cells was noticed by poly(ethylene oxide)-poly(propylene oxide)-poly(ethylene oxide) (PEO-PPO-PEO) nanoparticles (NPs) incorporated with kaempferol. Moreover, Poly (DL-lactic acid-co-glycolic acid) (PLGA) NPs incorporated with kaempferol showed an enhanced reduction in cancer cell viability, while no substantial reduction in cell viability of normal cells compared with kaempferol alone was observed. Consequently, both PEO-PPO-PEO and Poly (DL-lactic acid-co-glycolic acid) NP formulations were effective in reducing cancer cell viability [149]. Gold NPs (k-AuNPs) with kaempferol were made and their anti-cancer properties were evaluated. In the presence of k-AuNPs, a decrease in viability of MCF-7 breast cancer cells was noted as dose- and time-dependent. Moreover, k-AuNPs induced apoptosis of the cancer cells and they also inhibited the VEGF-induced angiogenesis [166]. 

The role of kaempferol loaded in nanostructured lipid carriers to increase cytotoxicity, efficiency, and paclitaxel-dependent apoptosis in breast cancer cells (MDA-MB 468) was investigated. The moderated cell proliferation from 56 ± 26.8% to 44 ± 3.9% was established by kaempferol-loaded nanostructured lipid carriers. Co-administration of kaempferol-loaded NPs as well as paclitaxel into cancer cells substantially strengthened the percentage of apoptosis. This result advocates that kaempferol incorporated into nanostructured lipid carriers as an anti-cancer adjuvant is an influential technique that might be a valuable delivery system to increase the efficacy of kaempferol as a chemotherapeutic agent on different cancer cells [168]. Kaempferol-conjugated gold nanoclusters (K-AuNCs) were made and their role as an anti-cancer drug was examined using A549 lung cancer cells. The prepared NPs mainly targeted and damaged the nuclei of the cancer cells. This nanocluster was less toxic to the normal human cells and showed greater toxicity to the A549 cancer cells [167]. Kaempferol-coated AgNPs caused cytotoxic effects and decreased the viability of HepG2 cells in a concentration-dependent way. Kaempferol-coated AgNP-treated cells showed an increased LDH leakage percentage. Kaempferol-coated AgNPs could also suppress the migrating as well as the invading ability of cancer cells (HepG2 cells), showing their anti-metastatic effect [169]. In vivo antitumor efficiency of PEGylated AuNPs-DOX@Kaempferol demonstrated a substantial decrease in tumor volume. The induction of apoptosis after the treatment with PEGylated AuNPs, DOX, kaempferol, PEGylated AuNPs-DOX, PEGylated AuNPs-Kaempferol and PEGylated AuNPs-DOX@Kaempferol for 24 h was assessed. Apoptotic cells increased significantly after the IC50 concentration of the PEGylated AuNPs-DOX@Kaempferol treatment compared to the others [170]. 

To evaluate the in vivo antitumor activity and to display body weight, 5 mg/kg of PEGylated AuNPs, DOX, kaempferol, PEGylated AuNPs-DOX, PEGylated AuNPs-Kaempferol and PEGylated AuNPs-DOX kaempferol was given orally. While treated with saline, the tumor volume was increased at 1066.17 ± 0.10 mm, demonstrating no significant tumor suppression. Followed by PEGylated AuNPs (900mm^3^), PEGylated AuNPs-DOX (700mm^3^) displays a moderate level of tumor growth suppression when compared to the control group. The PEGylated AuNPs kaempferol-treated group had a tumor volume of 500mm^3^ and the PEGylated AuNPs-DOX@Kaempferol-treated group had a volume of 270mm^3^ [170].

## 5. Kaempferol: Synergistic Effects in Combination with Anti-Cancer Drugs in Cancer Cells

**Figure 5:**
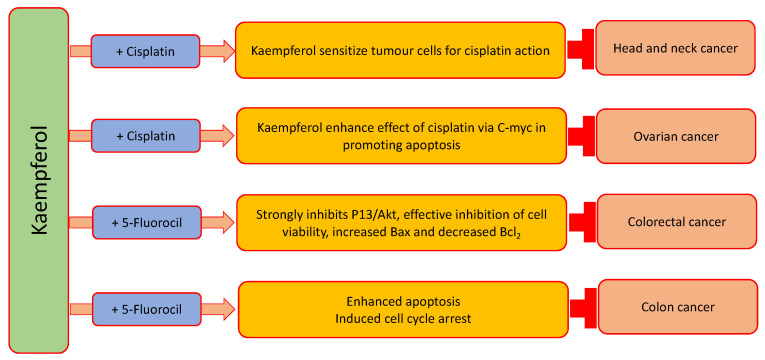
Synergistic effects of kaempferol in combination with 5-Fluorouracil inhibit cell viability, induce apoptosis, decrease bcl2, induce cell cycle arrest and inhibit PI3/Akt [161,162,163], and with cisplatin they increase CDKN1A mRNA, inhibit c-Myc mRNA and cell viability [165].

Natural compounds have also shown synergistic activity when they combine with anti-cancer drugs, as they enhance the efficacy of these drugs. Synergistic effects of kaempferol with cancer drugs have been described in earlier studies (Table 4) and (Figure 5). Anti-tumor effects were examined, and the probable mechanisms of kaempferol combined with 5-Fluorouracil in colorectal cancer cells were described. The combination of kaempferol and 5-Fluorouracil was recognized to be more powerful in blocking cell viability than either of the agents only. The inhibition of tumors in response to kaempferol and 5-Fluorouracil was related to the stimulation of apoptosis and the decrease in proliferation ability. The protein results designated that kaempferol and 5-FU might meaningfully downregulate the expression levels of Bcl-2 and TS and upregulate the expression levels of Bax. Additionally, the combination treatment significantly inhibited the PI3K/Akt pathway activation, signifying the involvement of this pathway in the synergistic effects [51]. Combined treatments with kaempferol and quercetin were more powerful than the additive effects of either of the compounds only. The reduction in cell proliferation was associated with decreased total protein levels and decreased expression of nuclear proliferation antigen Ki67 in treated cells relative to controls [171]. 

Kaempferol is capable of chemo-sensitizing 5-Fluorouracil-resistant LS174-R cells. Kaempferol combined with 5-Fluorouracil caused synergistic inhibitory effect on cell viability. Kaempferol combined with 5-Fluorouracil increased the apoptosis and induced cell cycle arrest of both chemo-resistant and sensitive cells via affecting the expression levels of various cellular effectors [172]. Kaempferol might sensitize head and neck tumor cells to the effects of cisplatin. These effects deliver novel confirmation for the use of a combination of kaempferol as well as cisplatin in vivo and their forthcoming applications in head and neck cancer therapy [173]. For kaempferol, which caused an important synergistic interaction with cisplatin, expression of ABCC6, NFkB1, cMyc, ABCC1, ABCC5 and CDKN1A genes was investigated. For cisplatin/kaempferol treatments on OVCAR-3 cancer cells, the mRNA levels of ABCC5, NFkB1 and ABCC1 did not change. Though, significant inhibition of ABCC6 as well as cMyc mRNA levels was seen for the cisplatin/kaempferol combined treatment. Cisplatin/kaempferol treatment upregulated the CDKN1A mRNA levels. Moreover, it was reported that kaempferol works synergistically with cisplatin in inhibiting cell viability of ovarian cancer, and their inhibition on cell viabilities was caused via ABCC6 and cMyc gene transcription inhibition [174]. 

## 6. Comparative Analysis of Kaempferol with Other Flavonoids/Natural Compounds

Flavonoids differing in the type as well as the number of substitution patterns exhibit different activity [175]. The effect of kaempferol and apigenin based on head and neck cancer cells was evaluated. Based on the results, the study demonstrated that at the lower doses (0.1 µM and 1 µM), kaempferol was more powerful than apigenin at decreasing cell viability. Though, at the higher doses (25 µM, 50 µM and 100 µM) and with 48 h of treatment, apigenin was more efficient than kaempferol in reducing cell viability. To determine whether apigenin and kaempferol would similarly alter cell viability, similar experiments were performed using PCI-13 and PCI-15B cells. The role of kaempferol in comparison to apigenin has been studied in different cell cancer lines such as Fa Du, PCL-13 and PCL-15 B. The results demonstrated that kaempferol showed a higher potential of killing cancer cells in Fa Du (49.7 ± 19.5 vs. 35.6 ± 18.5), PCL-13 (81.4 ± 2.2 vs. 46.2 ± 2.9) and PCL-15 B (118.78 ± 2.0 vs. 58.2 ± 3.0) as compared to apigenin [176].

Another study based on leukemia was performed to compare the molecular mechanism induced by kaempferol and EGCG in HL-60 leukemia cells. The results demonstrated that both compounds, kaempferol and EGCG, decreased cell viability and increased apoptosis in HL-60 cells. The activity of kaempferol in the reduction of cell viability and enhancement of apoptosis was more than EGCG [177].

The antitumor activity differences of kaempferol, kaempferol-7-O-glucoside (kae-7-O-glu), kaempferol-3-O-rhamnoside (kae-3-O-rha) and kaempferol-3-O-rutinoside (kae-3-O-rut) were analyzed. Kae-3-O-rha and kae-3-O-rut did not show effects on human hepatocellular carcinoma cell line HepG2 proliferation, whereas kaempferol and kae-7-O-glu (10–100 μM) caused a decreased proliferation with kaempferol showing the highest inhibitory effects. Additionally, in both human colon cancer cells CT26 and mouse melanoma cells B16F1, only kaempferol showed an inhibitory role, whereas the other three compounds did not show effects. To discover the differences in apoptosis-promoting capabilities, the effects of these four compounds on the expression of associated proteins in the apoptotic pathway were explored. The expression of cell apoptosis marker proteins was upregulated in kaempferol-treated cells. However, the other three compounds did not show noteworthy effects on apoptotic protein expression [178].

Comparative analysis of kaempferol and quercetin on the cytokine-caused pro-inflammatory status of cultured human endothelial cells was examined. The results revealed that the expression of adhesion molecules was more strongly prevented in kaempferol-treated than in quercetin-treated cells. The inhibitory potential on COX-2 and iNOS protein level was stronger for quercetin at 5–50 μmol/L. Further, the effect of kaempferol on NF-κB as well as AP-1 binding activity was weaker at high concentrations (50 μmol/L) as compared with quercetin [179].

## 7. Conclusions and Future Prospective

Cancer is the main culprit of death and its incidence is increasing day by day worldwide. Numerous treatment approaches are in practice to treat the cancer, but such treatment approaches cause various adverse effects. In this view, kaempferol, a natural flavanol, which is largely found in vegetables and fruits, has been revealed to have chemopreventive potential. Kaempferol acts through modulating cell signaling molecules including apoptosis, angiogenesis, autophagy, tumor suppressor genes, signal transducer and activator of transcription 3, telomerase, Nrf2, Ap1 and other cell signaling molecules. 

Some major challenges of kaempferol in health management include its very low aqueous solubility, rapid metabolism and lysosomal degradation and elimination from the body. Further, the potential role of kaempferol in cancer management is still to be investigated properly due to its poor bioavailability. Recently, nano-based formulations have been synthesized and their role in cancer has been described based on in vitro studies. Moreover, detailed studies should be performed based on in vivo to improve the bioavailability of this compound to explore its role in cancer management via a high concentration of this compound in tumor cells. Further, the synergistic effects of kaempferol with anti-cancer drugs have been described with greater efficacies through inhibition and activation of genes’ activities. A few studies based on human subjects have been conducted to evaluate its implication in health management and the role of kaempferol should be translated from preclinical to clinical cancer treatment approaches. 

Moreover, more studies and clinical trials in humans should be accomplished to discover the mechanism of action, safety, tolerability, the chemoprevention potential of kaempferol in humans and its finest beneficial dosage. Moreover, future challenges should be focused on novel nanoformulations being synthesized to overcome the bioavailability, rapid degradation and reduction in toxicity. This comprehensive review explained the anti-cancer potential of kaempferol based on in vivo and in vitro studies through modulation of various cell signaling molecules. The information compiled in this review will be helpful to the researchers to know in detailed means about the chemoprotective effects of this compound and its possible implication in clinical trials.

## Figures and Tables

**Table 1:** Mechanism of action of kaempferol in cancer prevention. Up-arrow (↑) indicates up-regulation and down-arrow (↓) indicates down-regulation.

Signaling Pathways	Cancer	Cell Line	Mechanism	Effects	Ref.
Angiogenesis	Cervical cancer	OVCAR-3 and A2780/CP70	*VEGF* gene expression and HIF-1alpha ↓	*VEGF* gene expression at mRNA and protein levels was decreased by kaempferol. Kaempferol treatment decreased HIF-1alpha (regulator of VEGF)	[41]
		OVCAR-3 and A2780/CP70	VEGF secretion ↓	Kaempferol inhibited VEGF secretion. Kaempferol decreased ERK phosphorylation, NFκB and cMyc expression	[31]
Apoptosis	Breast cancer	MB-453	p53 ↑	The kaempferol induced apoptosis and it was associated with the upregulation of p53	[32]
	Leukemia	MDA-MB-231	Caspase 9, 3 and p-ATM ↑	Kaempferol induced apoptosis and increased levels of γH2AX expression, cleaved caspase 9 and 3	[47]
		HL-60 and NB4	CASP3 and BAX/BCL-2 ratio ↑	Expression of CASP3 and BAX/BCL-2 ratio increased and BCL-2 decreased	[43]
Tumor suppressor gene	Bladder cancer	EJ	PTEN↑	Kaempferol enhanced PTEN expression. Kaempferol-induced apoptosis was partially attenuated in PTEN-knockdown cells	[33]
Autophagy	Liver cancer	SK-HEP-1	AVO-positive cells, auto phagosomes, autophagic cell death ↑	AMPK and AKT signaling pathways and CDK1/cyclin B expression contributed to kaempferol-induced death of autophagic cells and G2/M cell cycle arrest	[29]
	Gastric cancer	AGS, NCI-N87, SNU-638 and MKN-74	Autophagy and cell death ↑	Kaempferol activates the IRE1-JNK-CHOP signaling from cytosol to nucleus, and G9a inhibition activates autophagic cell death in cancer cells	[48]
hTERT pathways	Cervical cancer	HeLa	hTERT ↓	Kaempferol induced cellular apoptosis and aging via downregulating hTERT pathways	[49]
Signal transducer and activator of transcription 3 (STAT3)	Ovarian cancer	OVACAR-3	STAT3 ↓	Kaempferol induced cell cycle arrest of cancer cells at the G2/M checkpoint and inhibited the MEK/ERK and STAT3 signal transduction pathways	[34]
Transcription factor AP-1	Bone cancer	U-2 OS	DNA binding activity of AP-1 ↓	Kaempferol reduced the AP-1DNA binding activity	[35]
Nuclear factor E2-related factor 2 (Nrf2)	Lung cancer	A549 and NCIH460	Nrf2 mRNA and protein levels ↓	Kaempferol is a powerful inhibitor of Nrf2 and can be used as a natural sensitizer and anti-cancer agent for lung cancer therapeutics	[50]
Phosphatidylinositol-3-kinase (PI3K)/AKT	Colorectal cancer	HCT-8 or HCT-116	PI3K/Akt ↓	Synergistic effect of kaempferol with 5-Fluorouracil was noticed in the inhibition of cell proliferation and induction of apoptosis via suppression of thymidylate synthase or attenuation of p-Akt activation	[51]
ERK/p38 pathway	Ovarian Cancer	OVCAR-3 and SKOV-3	ERK1/2 and p38 ↑	Kaempferol sensitized ovarian cancer cells to TRAIL-initiated apoptosis through enhancement of DR4 as well as DR5 via ERK/JNK/CHOP pathways	[52]
Wnt/β-catenin signaling	Retinal tumor	SO-RB50	Wnt/β-catenin ↓	Kaempferol caused G2/M arrest as well as apoptosis and also suppressed Wnt/β-catenin signaling via targeting ERRα	[53]
Cell cycle	Breast cancer	MDA-MB-231	G1 phase ↓ Population of cells in the G2 phase ↑	After the treatment with kaempferol, the population of cells in the G1 phase was significantly reduced and the population of cells in the G2 phase was enhanced markedly	[54]
	Liver cancer	769-P and 786-O	Cells arrested at phase G2-M stage	After kaempferol treatment, most cells arrested chiefly at the phase G2-M stage	[37]
		K-HEP-1	G2/M cell cycle arrest	Kaempferol treatment resulted in G2/M cell cycle arrest	[29]

**Table 2:** Role of kaempferol in different cancers.

Types of Cancer	Cell Lines	Dose Range	Outcome of the Study	Ref.
Cervix cancer	HeLa cells	12–100 μM	Kaempferol decreased cell viability and increased cellular apoptosis	[49]
		20, 30 and 50 μM	Kaempferol treatment showed an increase in the proportion of apoptotic cells	[58]
Breast cancer	MDA-MB-231	50 Μmol/L	The population of cells in the G_1_ phase decreased from 85.48% to 51.35%, and the population of cells in the G2 phase enhanced with the kaempferol treatment.	[47]
	MDA-MB-231 and MDA-MB-453	10, 20, 40 μmol/L	Low dose of kaempferol meaningfully inhibited the migration of cancer cells	[122]
	MCF-7	100 and 30 µM	Kaempferol potently inhibited glucose uptake by breast cancer cells, essentially by reducing GLUT1-initiated glucose uptake	[123]
	MDA-MB-231, MCF-7 and T47D	20–100 µM	Kaempferol treatment caused a reduction in cell viability, an effect which occurred in a dose-dependent way	[124]
Ovarian cancer	A2780	40 µmol/L	Kaempferol decreased viability and increased cell apoptosis	[125]
	OVACAR-3	50 µM	Kaempferol (50 µM) significantly increased apoptotic cell percentage from 3.46% in control to 34.16% in cancer cells	[34]
	OVCAR-3 and SKOV-3	20–100 µM	Kaempferol increased apoptosis, whereas the expression of anti-apoptotic proteins declined	[52]
	A2780/CP70	0–80 μM	Substantially raised levels of caspase 3/7 noticed at 80 μM kaempferol for all cancer cell lines	[117]
Endometrium cancer	Ishikawa and HEC-265	36 and 72 μM	Kaempferol significantly induced apoptotic cell death. Additionally, kaempferol induced apoptosis mostly by inducing p53 and PARP	[126]
	MFE-280	0–20 μM	Kaempferol induced apoptotic cell death in the endometrial carcinoma cells in a dose-dependent way	[127]
Prostate cancer	LNCaP	5, 10, 15 μM	Kaempferol endorsed apoptosis of cancer cells. Based on in vitro study, kaempferol suppressed vasculogenic mimicry of cancer cells	[128]
Bladder cancer	EJ	20–80 μM	Expression levels of p-p53 and p-BRCA-1 increased, whereas the expression level of total p53 marginally increased	[129]
	5637 and T24	50–150 μM	PTEN increased in a time-dependent manner in cells treated with kaempferol while Ser473-phosphorylated Akt (pAkt) expression was significantly decreased	[33]
Renal cell carcinoma	786-O cell and 769-P cells	100 μM	After kaempferol treatment, maximum cells arrested mostly at phase G2-M stage, 52.36% in 786-O cells and 43.45% in 769-P cells	[37]
	RCC 786-O	0–150 μM	Kaempferol meaningfully decreased the invasion as well as migration of cancer cells	[121]
Liver cancer	SK-HEP-1	0, 50, 75, 100 μM	After kaempferol treatment, AVO-positive cells increased in a concentration-dependent way. Based on findings, it was concluded that kaempferol induced autophagy	[29]
	Huh7	0–100 μM	Exposure of Huh7 cells to kaempferol (10 μM) caused an important reduction in their viability	[130]
Gastric cancer	MKN28 and SGC7901	60 or 120 μM	Kaempferol led to a noticeable decrease in the protein levels of cyclin B1, Cdc25C and Cdk1 in a dose-dependent way	[118]
	AGS, SNU-216, NCI-N87, SNU-638, NUGC-3 and MKN-74	50 μM	Kaempferol meaningfully decreased cell viability in cancer cells and cisplatin in combination with kaempferol showed a multi-drug cytotoxic effect on cancer cells	[48]
Pancreatic cancer	Miapaca-2, Panc-1, and SNU-213	50 μM	Kaempferol precisely inhibited the cell viability by nearly 15% in Panc-1 cells, 40% in Miapaca-2 cells and 10% in SNU-213 cells	[131]
	PANC-1 and Mia PaCa-2	50 μM	Kaempferol significantly increased cancer cells’ apoptosis, and the rate of apoptosis was positively connected with kaempferol concentration	[67]
Bile duct cancer	HCCC9810 and QBC939	0–150 μM	Kaempferol significantly suppressed the viability of cancer cells in a time- and dose-dependent way	[132]
Gall bladder cancer	SGC996 and GBC-SD	0–200 μg/ml	Both the cancer cells were meaningfully arrested in the G0/G1 phase and showed a substantial decrease in the S as well as G2/M phases in a dose-dependent way	[133]
Oral cancer	SCC4	0–100 μM	Kaempferol substantially inhibited the migration and invasion of cancer cells in a dose-dependent way	[134]
Lung cancer	A-549	56 μM	Treatment of the cells with radiation only led to a marginal effect on clonogenic survival. Though, when pretreated with kaempferol before exposure to radiation, the surviving fraction decreased meaningfully	[120]
	A549 and NCIH460	1–50 μM	Kaempferol selectively decreased Nrf2 mRNA as well as protein levels	[50]
Bone cancer	U2	0–100 μM	Treatment of osteosarcoma cells with increasing concentrations of kaempferol reduced the cell invasion in a concentration-dependent way	[35]
	U-2 OS, HOB, 143B	25–200 µM	Kaempferol meaningfully decreased cell viabilities in a dose-dependent manner	[135]
Colon cancer	HT-29	0–60 μmol/L	Kaempferol increased DNA fragmentation, chromatin condensation and the number of early apoptotic cells in a dose-dependent way. Furthermore, kaempferol increased the levels of cleaved caspase-9, 3 and 7	[136]
	MSU2, HCT116, KNC	2.5, 5, 10, and 20 µM	Activation of phosphorylation of Stat3 and Erk was decreased by kaempferol	[137]
	SW480	10–40 µM	The combined treatment with kaempferol and tumor necrosis factor-related apoptosis-inducing ligand (TRAIL) radically induced apoptosis	[138]
Esophagus cancer	KYSE150 and Eca109	30–60 micro m	Kaempferol considerably inhibited tumor cell proliferation as well as clone formation	[119]
Skin cancer	A375	10–80 µM	Kaempferol showed powerful anti-cancer effects through apoptosis induction, cell migration inhibition, G2/M cell cycle arrest	[139]
	B16	50 μM and 100 μM	Kaempferol (50 μM and 100 μM) pointedly inhibited the cell viability of cancer cells	[140]
	A431, A431 sh-RSK2, A431 sh-MSK1, or A431 sh-RSK2/ sh-MSK1, NIH3T3	0–50 μM/L	Kaempferol relates to RSK2 as well as MSK1 at the ATP-binding pocket and prevents their corresponding kinase activities	[141]
Leukemia	THP-1	40 µM	Kaempferol and quercetin at a concentration of 40 µM reserved the proliferation of cancer cells	[142]
	Jurkat/Bcl-2/Jurkat/Neo	25–75 μM	Exposure of J/Neo cells to kaempferol showed activation of the ATM/ATR-Chk1/Chk2 pathway, cytotoxicity and triggered the phosphorylation of p53	[143]
	MOLT4	95 μM	Kaempferol plus TRAIL initiated apoptosis strongly in MOLT-4 cells after treatment	[144]
Fibrosarcoma	HT1080	10–100 μM	Kaempferol significantly decreased Matrix metalloproteinase-9 secretion and decreased transcription of Matrix metalloproteinase-9 mRNA	[145]

**Table 3:** Nanoformulation of kaempferol and its role in cancer.

Cancer	In Vivo/In Vitro	Nano Formulation	Outcome	Ref.
Ovarian	OVCAR-3	Poly (ethylene oxide)-poly (propylene oxide)-poly (ethylene oxide) (PEO-PPO-PEO) and Poly (DL-lactic acid-co-glycolic acid) (PLGA)	Both poly (ethylene oxide)-poly (propylene oxide)-poly(ethylene oxide) and Poly(DL-lactic acid-co-glycolic acid) nanoparticle formulations were effective in reducing cancer cell viability	[149]
Breast	MCF-7	k-AuNPs	k-AuNPs cause a decrease in viability and induce apoptosis	[166]
Breast	MDA-MB468	Kaempferol in Nanostructured Lipid Carriers (NLCs)	Kaempferol incorporated into nanoparticles as an anti-cancer adjuvant is an effective technique that may be a beneficial delivery system to increase chemotherapy agents	[168]
Lung	A549	Kaempferol-conjugated gold nanoclusters (K-AuNCs)	The synthesized nanoparticles chiefly targeted and damaged the nuclei of the cancer cells	[167]
Liver	HepG2	Kaempferol-coated sliver nanoparticles	Kaempferol-coated AgNPs caused cytotoxic effects and decreased the viability of cancer cells	[169]
Colon	HT-29/HT-29 tumor-bearing nude mice	Nanomaterial (PEGylated AuNPs-DOX@Kaempferol)	In vivo antitumor efficiency of PEGylated AuNPs-DOX@Kaempferol demonstrated a substantial decrease in tumor volume	[170]

**Table 4:** Synergistic effects of kaempferol with anti-cancer drugs/bioactive compounds.

Cancer Type	Cell Lines	Cancer Drugs/Bioactive Compound	Outcome	Ref.
Colorectal	HCT-8 or HCT-116	5-Fluorouracil	The combination of kaempferol and 5-Fluorouracil was recognized to be more powerful in blocking cell viability than either of the agents only	[51]
Breast	PMC42	Quercetin	Combined treatments with kaempferol and quercetin were more powerful than the additive effects of either of the compound only	[171]
Colon	LS174	5-Fluorouracil	Kaempferol combined with 5-Fluorouracil caused a synergistic inhibitory effect on cell viability	[172]
Head and neck		Cisplatin	Kaempferol may sensitize head and neck tumor cells to the effects of cisplatin.	[173]
Ovarian	OVCAR-3	Cisplatin	Kaempferol increased the effect of cisplatin via downregulation of cMyc in endorsing apoptosis of ovarian cancer cells	[174]

## Data Availability

Not applicable.
